# Microbial Communities Associated with Healthy and White Syndrome-Affected *Echinopora lamellosa* in Aquaria and Experimental Treatment with the Antibiotic Ampicillin

**DOI:** 10.1371/journal.pone.0121780

**Published:** 2015-03-20

**Authors:** David Smith, Peter Leary, Jamie Craggs, John Bythell, Michael Sweet

**Affiliations:** 1 School of Biology, Newcastle University, Newcastle upon Tyne, NE1 7RU, United Kingdom; 2 School of Biological Sciences, Medical Biology Centre, Queen’s University Belfast, Belfast, BT9 7BL, United Kingdom; 3 Horniman Museum and Gardens Aquarium, Forest Hill, London, SE23 3PQ, United Kingdom; 4 Biological Sciences Research Group, University of Derby, Kedleston Road, Derby, DE22 1GB, United Kingdom; Catalan Institute for Water Research (ICRA), SPAIN

## Abstract

Prokaryotic and ciliate communities of healthy and aquarium White Syndrome (WS)-affected coral fragments were screened using denaturing gradient gel electrophoresis (DGGE). A significant difference (R = 0.907, p < 0.001) in 16S rRNA prokaryotic diversity was found between healthy (H), sloughed tissue (ST), WS-affected (WSU) and antibiotic treated (WST) samples. Although 3 *Vibrio* spp were found in WS-affected samples, two of these species were eliminated following ampicillin treatment, yet lesions continued to advance, suggesting they play a minor or secondary role in the pathogenesis. The third *Vibrio* sp increased slightly in relative abundance in diseased samples and was abundant in non-diseased samples. Interestingly, a *Tenacibaculum* sp showed the greatest increase in relative abundance between healthy and WS-affected samples, demonstrating consistently high abundance across all WS-affected and treated samples, suggesting *Tenacibaculum* sp could be a more likely candidate for pathogenesis in this instance. In contrast to previous studies bacterial abundance did not vary significantly (ANOVA, F2, 6 = 1.000, p = 0.422) between H, ST, WSU or WST. Antimicrobial activity (assessed on *Vibrio harveyi* cultures) was limited in both H and WSU samples (8.1% ±8.2 and 8.0% ±2.5, respectively) and did not differ significantly (Kruskal-Wallis, χ2 (2) = 3.842, p = 0.146). A *Philaster* sp, a *Cohnilembus* sp and a *Pseudokeronopsis* sp. were present in all WS-affected samples, but not in healthy samples. The exact role of ciliates in WS is yet to be determined, but it is proposed that they are at least responsible for the neat lesion boundary observed in the disease.

## Introduction

Progressively more diseases of scleractinian corals are being reported in the scientific literature year on year [1–6]. Despite this, the causal agents of these diseases are often in dispute, with a single accepted pathogen for a given disease being rare [7]. This is the case for White Syndrome (WS), a disease commonly found in the Indo-Pacific and within aquarium systems [8–12]. WS is characterized by tissue loss and acute lesion progression with a distinct boundary or band between apparently healthy tissue and exposed skeleton [13].

In the past, WS has been attributed to various species from the gammaproteobacteria genus *Vibrio*, including; *V*. *harveyi* [14], *V*. *mediterranei* [7], *V*. *owensii* [15] and *V*. *coralliilyticus* [10]. Furthermore, Piskorska, Smith [7], reports an increase in *V*. *mediterranei* in the mucus of WS-affected *Echinopora lamellosa*, compared to healthy samples. However, Piskorska, Smith [7] used a culture-based method in determining bacteria associated with healthy and WS-affected *E*. *lamellosa*, an approach that can lead to phylum and class biases and result in inadequate profiling of microbial communities when not backed up with culture-independent techniques [16, 17], thus further work is required to confirm the role of *Vibrio* species in this disease.

Another proposed causal agent of WS includes *Arcobacter* sp, found by Sweet and Bythell [18] to be the only bacteria to be absent in non-diseased samples, appear in apparently healthy samples and increase to have a dominant presence in diseased *A*. *muricata* samples in a study screening the microbial communities associated with WS in this coral. Sweet and Bythell [18] also found *V*. *harveyi* to increase in diseased samples, but also to be present in healthy samples. This presence in healthy samples does not rule out *V*. *harveyi* as a primary causal agent in WS, as Sussman, Mieog [19] demonstrated that the zinc-metalloprotease (produced by pathogenic *Vibrio* sp) responsible for the breakdown of coral tissue and photo-inactivation of zooxanthellae is only expressed once *Vibrio* sp cell density exceeds 1x10^9^ cells ml^-1^. This indicates that *Vibrio* may not be harmful in lower densities, only becoming pathogenic as their numbers increase, explaining their presence in healthy samples and increase in diseased samples in the WS study by Sweet and Bythell [18].

The bacterial community associated with the holobiont of healthy corals is considered to play a potential role in sustaining coral health [20–24]. This community is known to shift in stressed corals, lowering coral defence and impeding important processes, such as the cycling of nitrogen and sulphur. Actinobacteria, for example, are known for their production of secondary metabolites which can be lethal to invasive bacteria [25–27]. Actinobacteria are also associated with healthy coral and have been found to decline in the event of environmental stress [28] and disease [7]. A shift in the natural microbial community associated with corals and a decline in actinobacteria could account for the proliferation of bacteria such as *Vibrio* sp, which would then become pathogenic in larger cell quantities [19] once their populations can no longer be controlled within the holobiont.

Only relatively recently have other microorganisms been studied with their relation to coral diseases. For example ciliates such as two scuticociliates from the genus *Philaster* have been isolated from WS-affected specimens of *Acropora muricata* and shown to be able to ingest the corals *Symbiodinium* and actively burrow into and underneath apparently healthy tissue in advance of the disease lesion [18, 29]. However, what hasn’t been proved to date is the exact role of these ciliates in coral disease and how many diseases suffer from ciliate infestations. Particularly, are the ciliates primary pathogens in WS and involved in the breakdown of host tissue, or alternatively are they secondary pathogens consuming dead and dying tissues. Additionally, the study by Piskorska et al. [7] did not investigate any potential ciliate community associated with WS in *E*. *lamellosa* and their roles in disease aetiology, as claimed necessary by Harvell, Jordán-Dahlgren [30].

This study aimed to determine the bacterial and ciliate communities associated with healthy and WS-affected *E*. *lamellosa* in aquaria, using culture-independent (16S rRNA gene) techniques. Additionally, the effect of experimental ampicillin treatment on the bacterial and ciliate communities of WS-affected *E*. *lamellosa* was analysed to determine the efficiency of antibiotic application in aquarium based corals and to infer whether certain bacteria are causal agents depending on their elimination from the bacterial community of treated samples.

## Methods

### Experimental Procedure

All coral samples used in this work were kindly donated by the Horniman Museum and Gardens Aquarium with permission of the senior curator for the aquarium, Jamie Craggs.

Both healthy and White Syndrome-affected *Echinopora lamellosa* fragments (donated by the Horniman Museum and Gardens Aquarium, London, UK) were placed into an aquarium tank system (26°C, 34 ppt salinity) at Newcastle University. Samples were characterised as apparently healthy (n = 3), untreated WS (n = 3) and treated WS (25 μg/ml ampicillin) (n = 3). Each sample type was placed into independent tanks to prevent contamination between different sample types during acclimatisation and thus prevent the spread of WS to healthy frags. Fragments were given 24 hours to acclimatise following transportation and disease progression was monitored regularly to ensure lesions were advancing in WS specimens before continuing with the study. Following acclimatisation, fragments were placed into their own independent tanks. These tanks were smaller in size and thus required much greater monitoring of water salinity and temperature. Since smaller tanks were not hooked up to the main inflow/outflow system, tank water was replaced daily with sterile salt water that had been passed through a 0.22μm filter using a sterile syringe. Ampicillin treatment was administered (every 4 hours) to n = 3 fragments with the aim of determining the causal agent/s of WS in *Echinopora lamellosa* through a process of elimination. Prokaryotic species present in untreated samples that were eliminated in treated samples (despite WS persisting in treated samples) would be unlikely to be causal agents of the disease.

Three samples exhibiting what appeared to be a lesion similar to that associated with WS were sampled immediately upon arrival at Newcastle University. These were characterized as “sloughed tissue” (ST) samples.

Samples were collected prior to complete tissue loss occurring in the WS-affected samples for microbial community analysis (stored in 100% ethanol at 4°C).

### DNA Extraction

To determine microbial community shifts in healthy (H), sloughed tissue (ST) untreated WS-affected (WSU) and treated WS-affected (WST) *E*. *lamellosa* samples, n = 3 H, n = 3 ST, n = 3 WSU, and n = 3 WST were analysed. Upon sampling, coral fragments were stored in 50 ml falcon tubes filled with 100% Ethanol and kept at 4°C until fragments were individually crushed using a sterilised pestle and mortar. DNA was extracted using the QIAGEN DNeasy Blood and Tissue Kit and resultant extracted DNA stored at 4°C.

### Prokaryotic 16S rRNA gene diversity

Using the universal prokaryotic primers, as used by Sweet et al, 2010; (357F) (5’-CCTACGGGAGGCAGCAG-3’) and (518R) (5’ATTACCGCGGCTGCTGG-3’), a segment of the bacterial 16S rRNA gene was amplified using a Hybaid PCR Express thermal cycler, prior to DGGE analysis. PCR cycles were carried out at 94°C for 30 seconds, 53°C for 30 seconds and 72°C for 1 minute and a total of 30 cycles were performed, as well as a final extension step at 72°C for 10 minutes (Sweet et al, 2010). PCR reaction mixtures were made up to 10 μl, using; 2 ng extracted DNA, buffer incubation mix with 1.5 mM MgCl_2_ (MP Biomedicals), 0.2 mM dNTP (Qiagen), 0.5 mM primer 357F, 0.5 mM primer 518R, 2.5 U of DreamTaq proof-reading DNA Polymerase (Fermentas). Each PCR sample mixture was done in triplicate and resulting PCR products combined to bring about a final volume of 30 μl for each sample. PCR products were verified by agarose gel electrophoresis [1% (w/v) agarose] with ethidium bromide staining and visualised using a UV transilluminator.

DGGE was performed using the D-code universal mutation detection system (Bio-Rad). PCR products were resolved on 10% (w/v) polyacrylamide gels using a 30 to 60% denaturant gradient for 16 hours at 60°C, with a constant voltage of 50 V. Following DGGE, gels were stained by pouring over a combined solution of 9 μl SYBR Gold (Sigma-Aldrich) and 50 μl 1xTAE buffer, and then left covered in the dark for 20 min. Gels were then washed in 500 ml 1xTAE buffer for 30 min and visualised using a UV transilluminator. To identify bands of most interest in the DGGE gels (those that represented the greatest differences/similarities between samples), representative bands were excised from the gel, left overnight in Sigma molecular grade water and then re-amplified using primers 357F and 518R. The products of each re-amplified band were then verified by agarose gel electrophoresis [1% (w/v) agarose] with ethidium bromide staining and visualised using a UV transilluminator. PCR products were then purified using the QIAGEN PCR Purification Kit (see main methods section for complete description) and then labelled using a Big Dye (Applied Biosystems) transformation sequence kit and sent to Genevision (University of Newcastle) for sequencing. Sequences were then compared with sequences in the BLAST nucleotide database (NCBI) to allow for genus/species matching.

Using Bionumerics 3.5 (Applied Maths BVBA) software, bacterial operation taxonomic units (OTUs) were defined from DGGE band-matching analysis. Standard internal marker lanes were used to allow for gel-to-gel comparisons. Tolerance and optimisation for band-matching was set at 1%.

### Ciliate 18S rRNA diversity

Ciliate-specific 18S rRNA gene amplification was carried out using the same ciliate primers as Sweet and Bythell [18]; forward primer CilF (5’-TGGTAGTGTATTGGACWACCA-3’) with a 36 bp GC clamp [31] attached to the 5’ end and the reverse primer CilDGGE-r (5’-TGAAAACATCCTTGGCAACTG-3’). PCR reaction mixtures were made up to 10 μl volumes, using; 20 ng extracted DNA, buffer incubation mix T.pol with 1 mM MgCl_2_ (MP Biomedicals), 100 μM dNTP (Qiagen), 0.2 μM 357F, 0.2 μM 518R, 1 U of DreamTaq DNA Polymerase (Fermentas). PCR was carried out using a Hybaid PCR Express thermal cycler. PCR amplification was carried out using an un-nested approach [32], with primary denaturation being carried out at 94°C for 5 min, followed by 26 PCR cycles of 94°C for 1 min, 52°C for 1 min and 72°C for 1 min. A final elongation step at 72°C was then carried out for 10 min to reduce heteroduplexes in the DGGE [33]. Each PCR reaction was carried out in triplicate and the resulting PCR products combined to bring about a final volume of 30 μl for each sample. PCR products were verified by agarose gel electrophoresis [1% (w/v) agarose] with ethidium bromide staining and visualised using a UV transilluminator. DGGE was performed using the D-code universal mutation detection system (Bio-Rad). PCR products were resolved on 6% (w/v) polyacrylamide gels possessing a 32 to 42% denaturant gradient for 16 hours at 60°C, with a constant voltage of 50V. Gels were stained and DGGE bands excised and purified as above, but using the ciliate primers. Samples were sent to Genevision (Newcastle University) for sequencing. DGGEs and samples were analysed as above. Retrieved ciliate nucleotide sequences have been submitted to Genbank with the following accession codes: KP793000 (C1), KP793001 (C2), KP793002 (C3) and KP792999 (C4).

To confirm the presence of *Philaster* sp. in diseased samples and clear up any ambiguity from the DGGE, a PCR product check was carried out on all samples using the primers BrB-F-171 (5’-TCAAACCCGACTTTACGGAAG-3’) and BrB-R-1721 (5’-TGCAGGTTCACCTACGGAAAC-3’) [34]. These primers were designed to amplify the ciliate associated with Brown Band Disease identified by Bourne et al. [34] and since described for WS by Sweet and Bythell [18], but also amplify a range of similar *Philaster* species and close relatives. PCR mixes were as described for the amplification of ciliate 18S rRNA in DGGE. PCR amplification was carried out at 95°C for 3 minutes, followed by 35 PCR cycles of 95°C for 30 s, 45°C for 45 s and 72°C for 2 min. A final elongation step was carried out at 72°C for 10 min [34]. Product checks were carried out using gel electrophoresis on a 1% agarose gel.

### Total Bacterial Count

To assess total bacterial abundance, 700 μl of each sample was vacuum-filtered through a 0.22 μm black polycarbonate filter and then stained with 100 μl of 4% PBS (phosphate buffer saline)-buffered paraformaldehyde solution containing 4’6-diamidino-2-phenylindole (at a final concentration of 5 μg ml^-1^) for 10 min. The filter was then rinsed with 1 x PBS pH 7.4 [35]. The stained filter was mounted onto a microscope slide and viewed under fluorescence microscopy, under which 50 images were taken of each sample. The bacteria present in each image were counted using the automated cell counter; Cell C [36, 37]. Parameters were set to exclude any objects within an image that were smaller than 0.0314μm^2^ and bigger than 0.6188μm^2^. Mean abundance of all 50 images per sample was then compared across all samples.

### Antimicrobial Properties against *Vibrio harveyi*

The inhibition of bacterial growth was determined from the ethanol-soluble fraction of apparently healthy and WS-affected *E*. *lamellosa* samples. Each tissue sample was transferred into sterile pre-weighed eppendorfs and then dehydrated in a vacuum centrifuge to remove any ethanol in which the coral fragments were stored. Each eppendorf was then reweighed to determine the dry weight of tissue and 100% ethanol added to make all samples reach a standard concentration of 100 mg/ml, to be used as a stock solution in testing bacterial inhibition by coral extracts.

Pure cultures of *Vibrio harveyi*, a bacterial species heavily considered the causal agent of WS, was isolated from an aquarium tank that had previously housed WS-affected corals. *V*. *harveyi* was isolated using thiosulfate-citrate-bile-sucrose (TCBS) *agar* and then subsequently grown in LB broth overnight (at 28°C) to get a fresh stock culture to be used in each assay. The concentration of bacterial culture to be used in each assay was then standardised to 0.15 optical density (OD) units by diluting the primary stock with additional sterile LB broth accordingly.

Antimicrobial assays were conducted in 96-well plates containing a triplicate control for uninhibited *V*. *harveyi* growth (97.5 μl Mueller Hinton Broth + 97.5 μl of *V*. *harveyi* culture), a triplicate control for the effect of 100% ethanol (97.5 μl MHB + 5 μl 100% ethanol + 97.5 μl *V*. *harveyi* culture) and a triplicate of each sample (97.5 μl MHB + 5 μl coral extract + 97.5 μl of *V*. *harveyi*). Using 5 μl coral extract in a 200 μl volume mix brought the final working concentration of coral extract to 2.5 mg/ml for each sample, as conducted by Sweet et al. [28].

The kinetics of bacterial growth was determined by reading the OD every 5 minutes over a 24 hour period in a Biotek Power Wave HT plate reader. The rate of bacterial growth during exponential phase was estimated by plotting OD against time to determine the gradient of each curve in a linear regression. Inhibition was then estimated by dividing the mean gradient of each sample type over the mean gradient of the ethanol controls. This was then calculated as % inhibition of bacterial growth when compared with ethanol control growth.

### Statistical Analysis

For DGGE profiles, a pairwise ANOSIM based on Bray-Curtis indexes were performed to determine differences between gene assemblages associated with the different coral samples (H, ST, WSU and WST). In order to determine which bacterial ribotypes showed the greatest shifts in presence between sample types, similarity percentages (SIMPER) analyses were conducted. All statistical analyses on bacterial DGGE profiles were conducted using PRIMER V. 6 software [18, 38, 39].

As data was found to be normally distributed and treatment variances found to be approximately equal, bacterial abundance in *E*. *lamellosa* samples was compared across all sample types with a one-way ANOVA. A Tukey HSD test was further conducted to determine pairwise differences between particular samples.

A Levene’s test for homogeneity showed that variances were not equal across treatments for percentage growth inhibition, even after the data had been transformed using a square-root transformation. A Kruskal-Wallis test was therefore carried out instead to determine differences in *Vibrio harveyi* growth inhibition between coral samples.

## Results

### Prokaryotic 16S rRNA gene diversity

**Fig 1:**
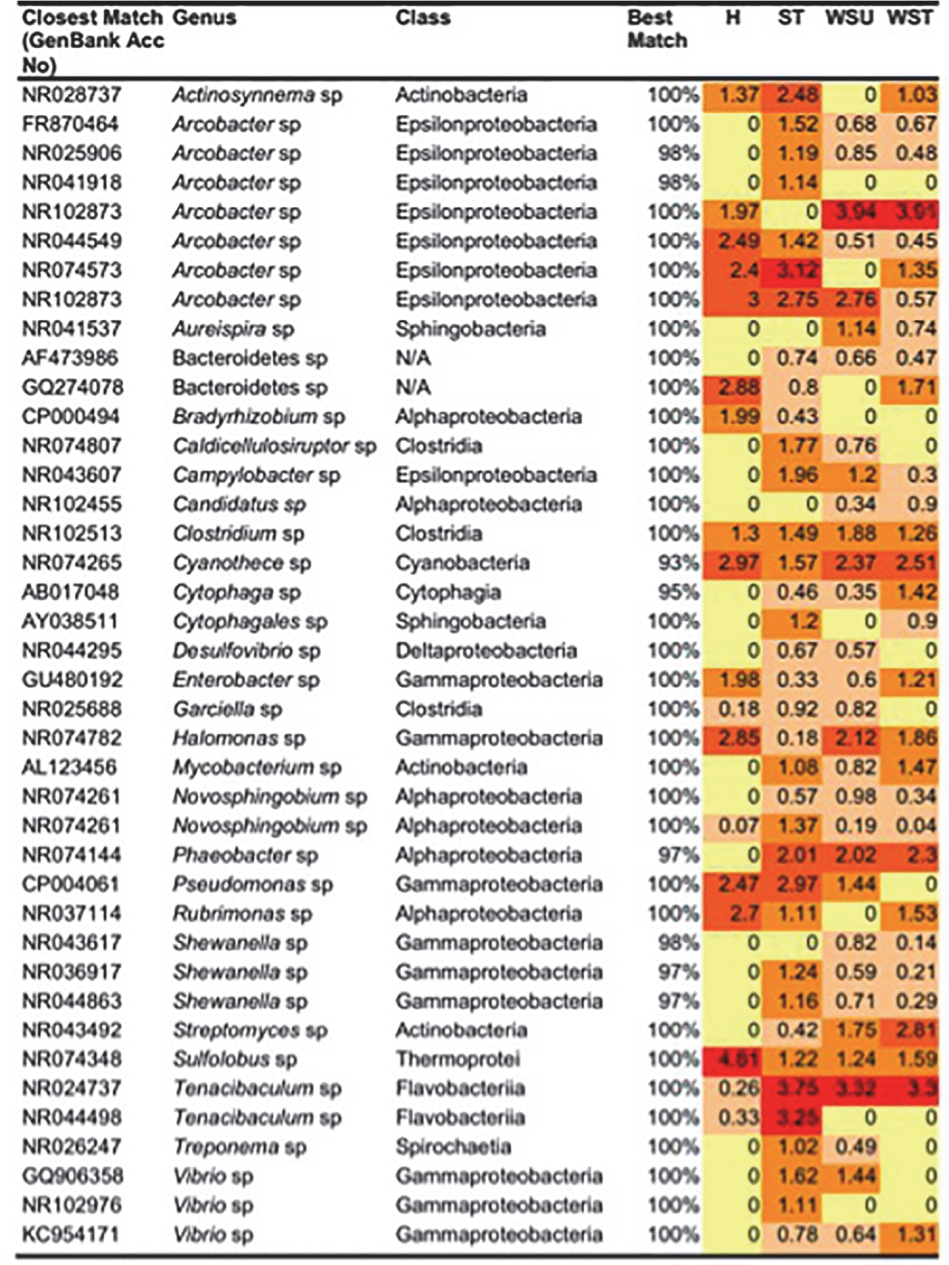
Heat-map representing the relative abundance of identified (GenBank ID) bacteria and archaea taxa associated with H, ST, UWS and TWS samples. Identification and sequence similarity was based on 161 bp sequences obtained from excised denaturing gradient gel electrophoresis (DGGE) bands. Relative abundance values are of scaled arbitrary units, based on relative density of DGGE bands in BioNumerics (3.5) analysis. Absence of a particular band is represented by a zero.

A one-way ANOSIM revealed a significant difference (R = 0.907, p < 0.001) in the bacterial/archaeal community profiles associated with healthy, sloughed tissue, untreated white syndrome (WS) and treated WS-affected *Echinopora lamellosa* samples (based on DGGE band matching analysis). Altogether, a total of 40 different 16S rRNA ribotypes were successfully determined from study samples (Fig. 1). Prokaryotic diversity was found to be greater in WS-affected samples than in healthy samples, with 37 identified prokaryotes being found in ST samples, 31 in UWS samples, 30 in TWS and 18 in H samples. Many bacterial ribotypes were detected in WS-affected samples, but found to be absent in healthy tissues thus their grouping as WS-associated bacteria. The most dominant of these ribotypes includes ones most closely related to; 3 *Vibrio* species. (GenBank accession numbers; GQ906358, NR102976 and KC954171), 3 *Arcobacter* species (NR041918, FR870464 and NR025906), 2 *Shewanella* species (NR036917 and NR044863), a *Mycobacterium* species (AL123456), a *Campylobacter* species (NR043607), a *Caldicellulosiruptor* species (NR074807), a *Treponema* species (NR026247), a *Cytophaga* species (AB017048), a *Streptomyces* species (NR043492) a *Novosphingobium* species (NR074261) and a *Phaeobacter* species (NR074144). Ribotypes closely related to Two *Tenacibaculum* species (NR024737 and NR044498) and a *Garciella* species (NR025688) were also found to markedly increase in all ST and WSU samples, compared to healthy samples (Fig. 1). Since ampicillin treatment failed to stop lesion progression, WS-associated ribotypes of particular interest were those that were still dominant across all samples after the antibiotic treatment. These included ribotypes related to; 3 *Arcobacter* species (FR870464, NR025906 and NR102873), a *Mycobacterium* species (AL123456), a *Vibrio* species (KC954171), a *Phaeobacter* species (NR074144), a *Tenacibaculum* species (NR024737), a *Streptomyces* species (NR043492), and a *Cytophaga* species (AB017048). Of these, only ribotypes closely related to the *Tenacibaculum* species and the *Phaeobacter* species were found to be consistent across all ST, WSU and WST samples. 16S rRNA ribotypes related to a *Sulfolobus* species (NR074348), a *Rubrimonas* species (NR037114), an *Arcobacter* species (NR044549), a *Bradyrhizobium* species (CP000494), an *Enterobacter* species (GU480192) and a Bacterioidetes species (GQ274078) were found to be reduced or absent in all ST and UWS samples compared to healthy samples, with only the latter two ribotypes, (related to the *Enterobacter* species and the Bacteroidetes species) being re-established following ampicillin treatment (Fig. 1). Due to their association with healthy *E*. *lamellosa* samples, these bacteria were considered to potentially have a beneficial role within the holobiont. Two dominant ribotypes, similar to a *Clostridium* species (NR102513) and a *Cyanothece* species (NR074265), were present across all samples, maintaining the same relative abundance throughout (Fig. 1).

### Bacterial Abundance

**Fig 2:**
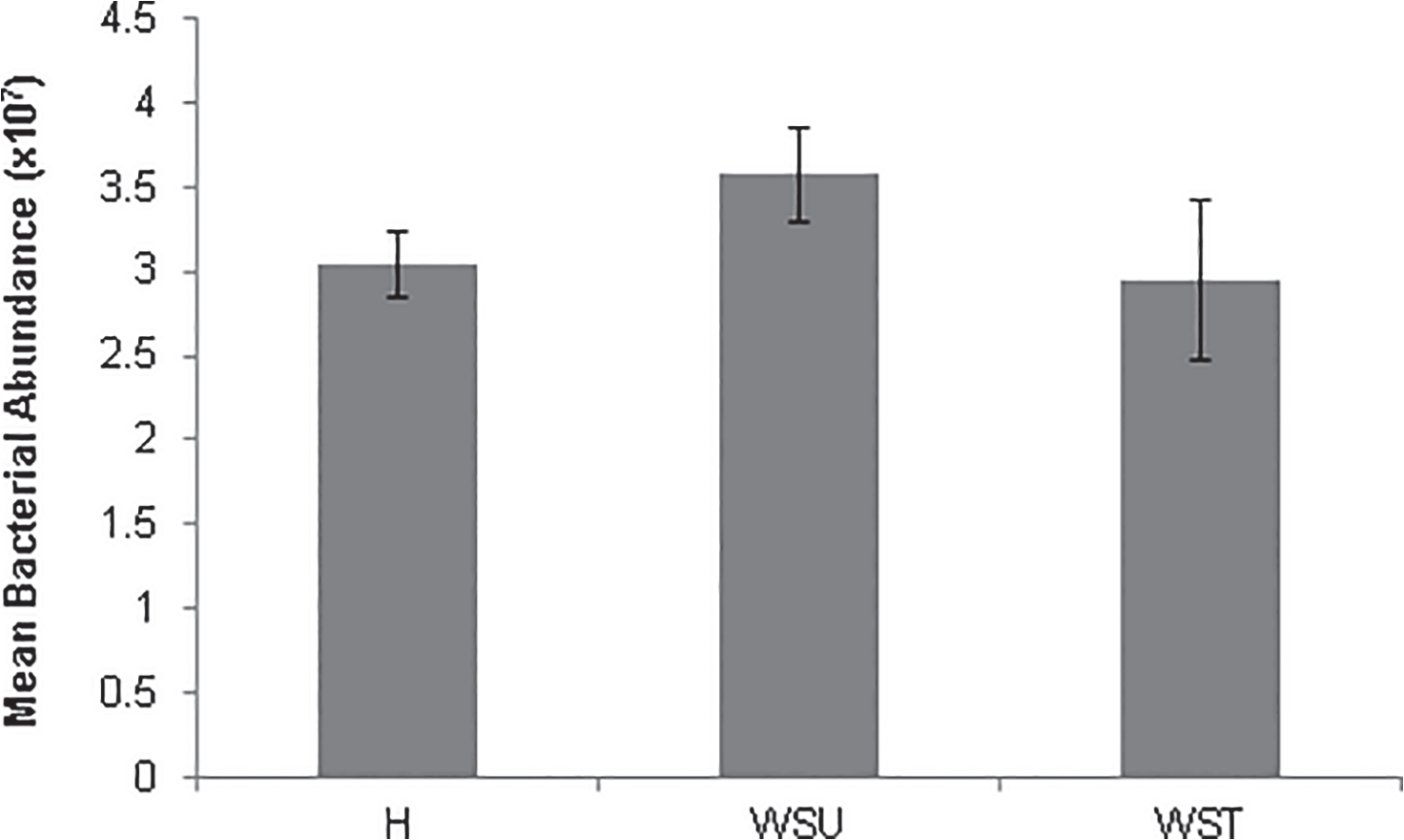
Bacterial abundance of *Echinopora lamellosa* samples. Mean bacterial abundance for apparently healthy (H), untreated White Syndrome-affected (WSU) and treated White Syndrome-affected (WST) *Echinopora lamellosa* samples. Error bars represent SE.

Total bacterial abundance did not differ significantly between samples (ANOVA, F_2, 6_ = 1.0, *p* = 0.422). Despite the lack of significant difference found in the one-way ANOVA, Bacterial cell count was found to be lower by ∼5x10^6^ cells in apparently healthy and ampicillin treated WS-affected samples compared to untreated WS-affected samples (Fig. 2).

### Antimicrobial Properties

**Fig 3:**
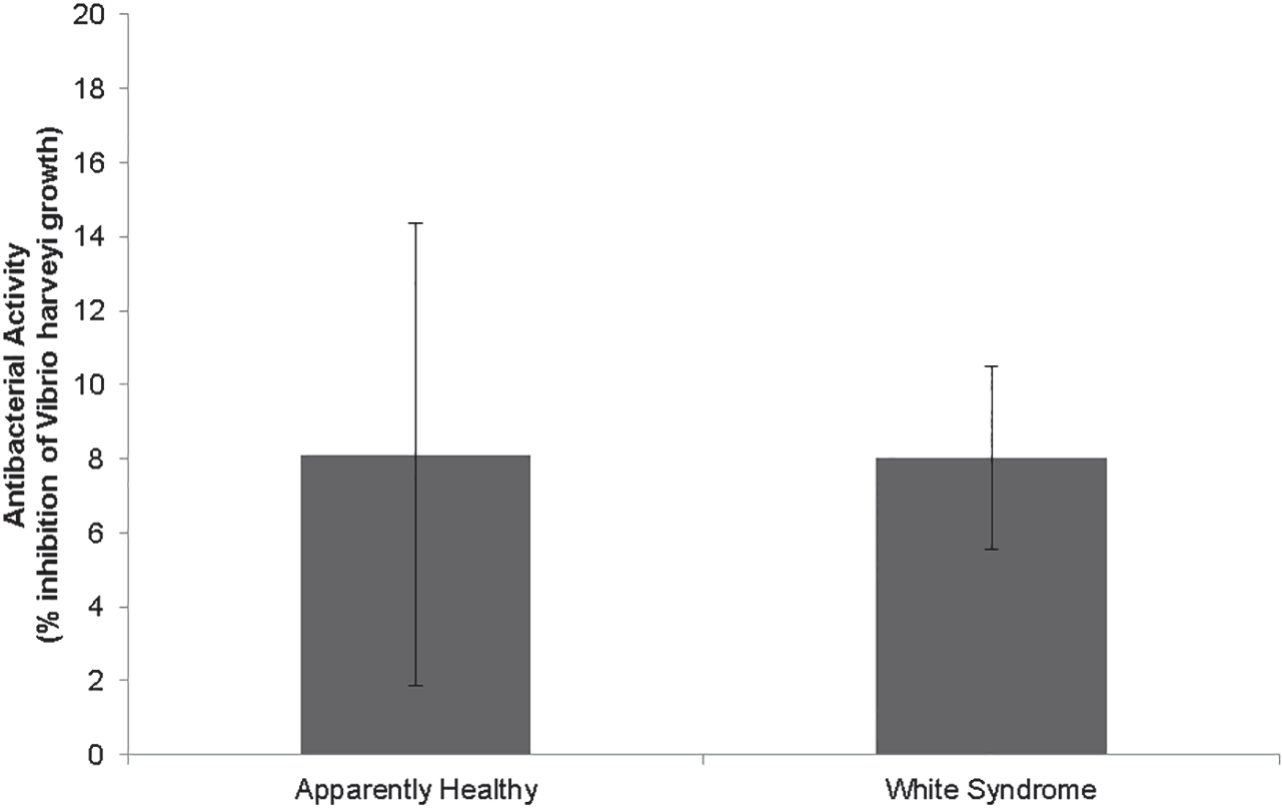
Antimicrobial activity of apparently healthy and White Syndrome-affected *Echinopora lamellosa* samples.

There was no significant difference (Kruskal-Wallis, χ^2^_(2)_ = 3.842, *p* = 0.146) in the median growth rate of *Vibrio harveyi* between apparently healthy and WS-affected samples. Percent growth inhibition of apparently healthy and WS-affected samples was found to be 8.1 ±8.2% and 8.0 ± 2.5%, respectively (Fig. 3). This indicates that apparently healthy and WS-affected *E*. *lamellosa* samples have very little or no antimicrobial capacity to limit *V*. *harveyi* growth.

### Ciliate 18S rRNA gene diversity

**Fig 4:**
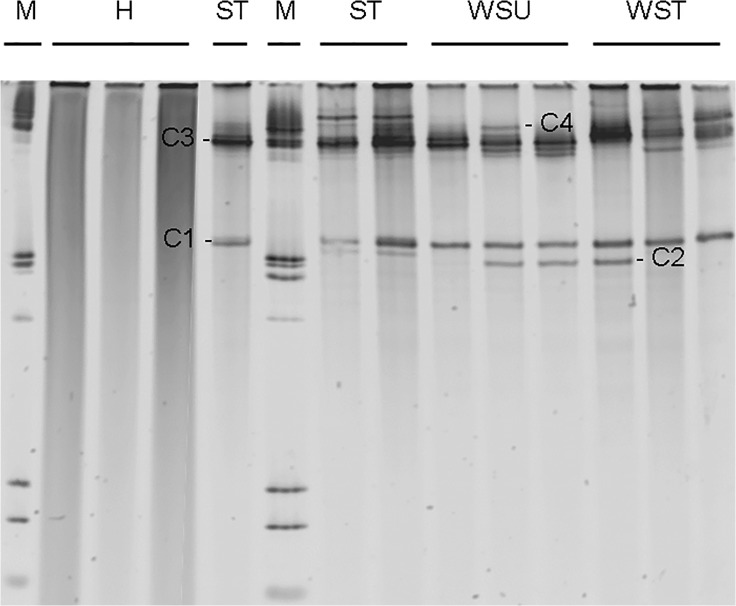
Ciliate 18S rRNA analysis of *Echinopora lamellosa* samples. Ciliate 18S rRNA gene fingerprints (represented on Denaturing Gradient Gel Electrophoresis) of healthy (H), sloughed tissue (ST), untreated (WSU) and treated WS (WST) samples. M = marker lane.

**Fig 5:**
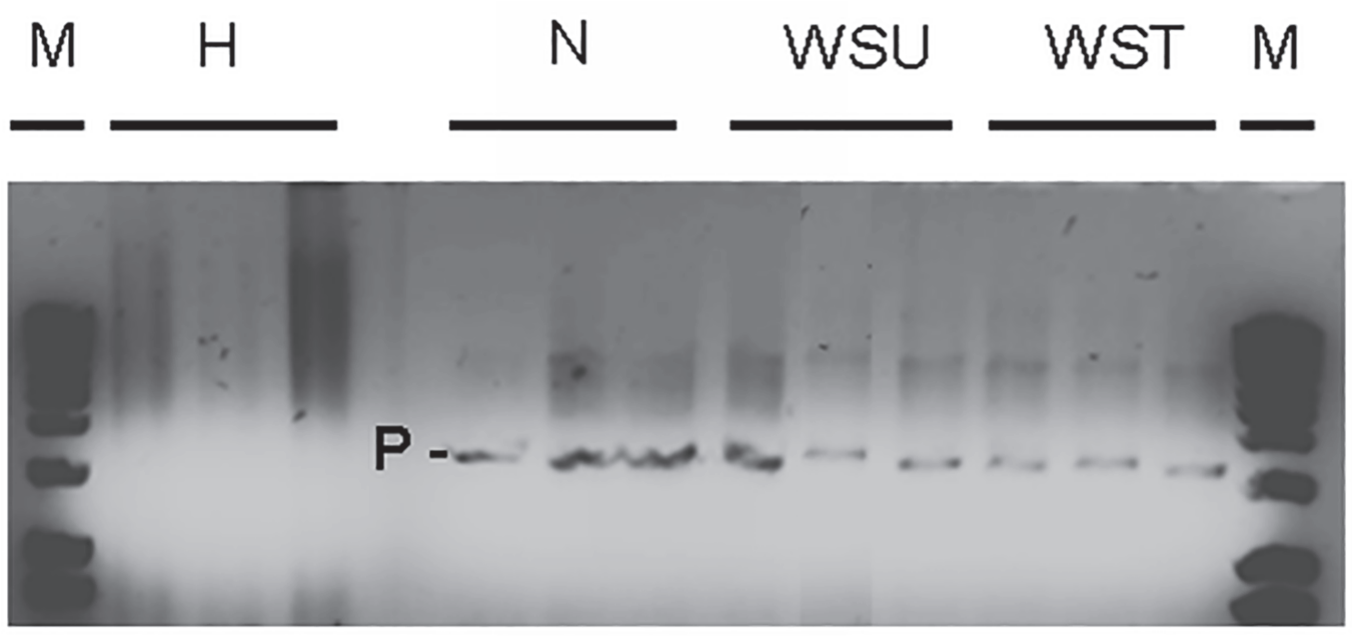
Determination of *Philaster* sp presence. PCR product check using *Philaster* sp specific primers (BrB-F-171 and BrB-R-1721) on healthy (H), sloughed tissue (ST), untreated (WSU) and treated WS (WST) samples, carried out using gel electrophoresis on a 1% agarose gel. P indicates bands representing *Philaster* sp. presence.

**Fig 6:**
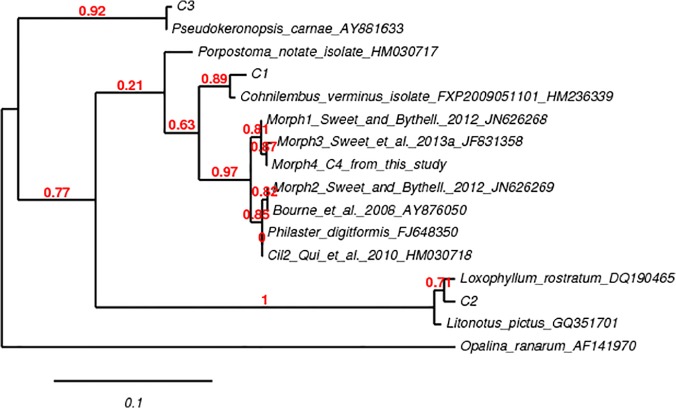
Neighbour-joining consensus tree of partial 18S rRNA gene sequences of 4 ciliate species found in *Echinopora lamellosa* samples affected by White Syndrome in this aquarium experiment. Sequence alignment was carried out using MUSCLE and the neighbour-joining tree was constructed in TreeDyn (via phylogeny.fr), using the Tamura genetic distance model [40] with an opalinid protist; *Opalina ranarum* (AF141970) as the outgroup, as used by Sweet and Bythell [18]

The ciliate 18S rRNA DGGE profile (Fig. 4) demonstrated the presence of several ciliate species in all white syndrome-affected *E*. *lamellosa* samples (treated and untreated), with no ciliates found in healthy samples, indicating the unique association of ciliates with WS pathology. Ciliates appear to be unaffected by treatment with ampicillin, as the ciliate community 18SrRNA gene profile appears relatively unchanged across all ciliate-infected samples (Fig. 4). Ciliate ribotypes present in diseased samples included those most closely related to: *Cohnilembus* sp. (C1, GenBank accession number Z22878), *Loxophyllum* sp. (C2 DQ190465), *Philaster* sp. (C4, JF831359) *and Pseudokeronopsis* sp. (C3, AY881633). Ciliates C1 (97% similarity to *Cohnilembus verminus*) and C3 (99% similarity to *Pseudokeronopsis* sp) appear to be present across all WS-affected samples (treated and untreated) whilst there is more ambiguity over the presence of C4 across all samples (with definite bands only appearing in one WSU and one WST sample) on the DGGE profile (Fig. 4). A PCR product check using *Philaster* sp. specific primers confirmed the presence of C4 in all ST, WSU and WST samples, with an absence in all H samples (Fig. 5). C4 was more closely related to the more ciliates associated with WS than with Brown Band Disease (Fig. 5). Moreover, C4 was found to be most closely related to *Philaster digitformis* Morph 3 (Fig. 6), a ciliate isolated from aquarium WS by Sweet, Craggs [29]. C4 differed from Morph 3 by 1 base pair over 445 bases, thus C4 is proposed to be a new morph of *P*. *digitformis*, designated as Morph 4. Morph 4 differs from the wild-type WS Morph 1 [18] by 2 base pairs over 445 bases. C2 (98% similarity with *Loxophyllum rostratum*) is only represented in two WSU and one WST sample on the ciliate 18S rRNA DGGE profile (Fig. 4). A lack of consistency in this latter species indicates that it is unlikely to be involved in the aetiology of WS.

## Discussion

This study is the first to simultaneously investigate the bacterial, archaeal and ciliate diversity of healthy and White Syndrome (WS)-affected samples of the scleractinian coral *Echinopora lamellosa*, whilst also determining WS-associated microbial diversity shifts in response to antibiotic treatment (using the antibiotic ampicillin). The bacterial and archaeal (16S rRNA) diversity associated with *E*. *lamellosa* was found to increase between healthy and WS-affected samples, indicating that various species will opportunistically enter the holobiont [41] or newly exposed niches (such as the coral skeleton) when a coral is diseased [18]. Only two bacterial ribotypes were found to increase considerably in 16S rRNA gene DGGE profiles between all healthy and WS-affected samples and maintain this DGGE band density in ampicillin-treated samples: a sequence related to a *Tenacibaculum* species and one related to a *Phaeobacter* species (with the former being represented by a weak DGGE band in healthy samples and the latter being absent in healthy samples). *Tenacibaculum maritimum* has previously been shown to be associated with a variety of different organisms in the marine environement, specifically associated with mucus layers such as in fish [42] and the soft coral *Dendronephthya* sp, in which the bacterium has been shown to produce a biofilm on the coral surface [43]. *T*. *maritimum* has also been shown to be responsible for an ulcerative disease known as tenacibaculosis, which affects the body surface of fish, resulting in the formation of ulcers, lesions and tissue necrosis on their surfaces [42, 44]. Given the pathogenicity of members of this genus, it is possible that the bacterium detected in this study (which increased dramatically in WS samples) is carrying out a similar role. Furthermore, this genus has previously been found to increase in potentially stressed and tumour-affected soft tissue in *Echinopora lamellosa*, which often subsequently became affected by WS [45], as well as having recently been associated with WS in *Acropora cervicornis* [46]. Interestingly, the ribotype related to *Phaeobacter* spp has in contrast, previously been found to provide protection to certain hosts (specifically fish and mollusc larvae) against pathogenic bacteria such as common coral associated bacteria such as the *Vibrio* spp. They do this by production of the antagonistic compound tropodithietic acid (TDA) [47–50]. Furthermore, *Phaeobacter* sp. has also previously been found in associated with *Oculina patagonica*, from which isolates showed antimicrobial action against the coral pathogens *Vibrio shiloi*, *V*. *coralliilyticus* and *Thalassomonas loyana* [22]. It could therefore be expected that *Phaeobacter* spp should aid in coral defence, the increase of this ribotype in WS samples could be an immune response initiated by the coral or the bacteria itself.

Several species of *Vibrio* [7, 10, 14, 15] and an *Arcobacter* sp [18] have been proposed as causal agents for WS, with species from both of these genera being identified in WS samples in this study. Sussman, Mieog [19] have previously demonstrated that *Vibrio* spp require a population of 1x10^9^ in order for proteases involved in virulence to be produced, thus if a *Vibrio* sp is the cause of WS in this study, complete dominance across all disease and treated samples would be expected, as stated by Sweet, Craggs [29]. No *Vibrio* sp were found in healthy samples, with all species being associated with WS-affected samples. Only one of these species, however, was found to be present in *E*. *lamellosa* samples treated with the antibiotic ampicillin (yet it was only detected in two out of three WST samples). Four of the seven *Arcobacter* sp found in this study were associated with healthy samples in relatively high abundance, with only one of these species increasing considerably in diseased samples (yet one *Arcobacter* sp also declined in response to the onset of WS). The other three identified *Arcobacter* sp. were found to exist inconsistently in WS-affected samples or in limited abundance. Using a process of elimination, the results of this study suggest that *Vibrio* sp and *Arcobacter* sp are not the causal agent for WS. An alternative view point may be that they are the causal agent for some WS outbreaks but not others, with WS being a polymicrobial disease caused by any opportunistic pathogenic bacteria at any given time. If the latter is true, then this leads to two possible scenarios for WS: a) WS is not a single disease, but rather different diseases that appear morphologically similar, or b) WS is the result of a systemic infection which can be caused by various pathogenic bacteria [29].

Over recent years, members of the coral holobiont have been found to provide particular services to their host, carrying out functions necessary for maintaining coral health, such as the cycling of nitrogen and sulphur and the production of secondary metabolites with an antimicrobial action useful against invasive bacteria [20–24, 51]. Based on the potential importance of various holobiont members, the loss of particular bacteria or archaea could have an impact on coral health. Three species identified in this study (closely related to a *Bradyrhizobium* sp., a *Rubrimonas* sp. and a *Sulfolobus* sp.) were found to decline considerably between healthy samples and WS-affected samples, with their populations failing to recover to healthy levels in response to treatment (with the prokaryote closely related to *Bradyrhizobium* sp. being completely lost). *Sulfolobus* spp. are known to be sulphur oxidisers [52, 53], converting sulphur (S_8_) into sulphate (SO_4_^2−^), thus a depletion of *Sulfolobus* spp. within the local microenvironment could result in an increase in sulphur and a decline in sulphate, provided there aren’t other members of the holobiont that can persist and carry out this function. An increase in sulphur ions can result in tissue necrosis in corals, as associated with Black Band Disease [54] and sulphate reduction could result in reduced DMSP output by zooxanthellae, a molecule which Raina, Tapiolas [55] state could have a significant role in antioxidant and antimicrobial production, osmoprotection and the shaping of the microbial community within the holobiont. *Bradyrhizobium* spp are denitrifying bacteria known to completely denitrify nitrates to nitrites to nitrogen gas, thus a reduction in this species could impact on the cycling of nitrogen within the microenvironment, resulting in a more nitrogen-limited environment. Nitrogen-limitation has been found to directly impact zooxanthellae, resulting in a decline in growth and population density [56].

Bacterial abundance did not differ significantly, implying that in this particular case, WS is not the result of a general increase in bacterial load. This is in contrast to previous studies such as Luna, Biavasco [57] and Sweet, Craggs [29] whereby bacterial abundance was found to increase in other aquarium corals exhibiting WS. Since treatment with ampicillin impacted bacterial diversity, yet overall bacterial abundance was not found to decrease in treated samples, it is possible that there was a very rapid colonisation by bacteria once the coral was replaced back into the tank system. However, Sweet, Croquer [37] showed that bacterial populations took >12 hours to recover under field conditions following treatment of healthy corals with Ciprofloxacin, which suggests that such rapid recolonisation is unlikely. The Coral Probiotic theory [21] proposes that the natural coral holobiont is host to microorganisms that aid in coral health and defence against invading bacteria via the production of secondary metabolites. This defence may be lost during an episode of environmental stress or disease due to a re-shuffling of the microbial community [21] or in this instance disease and subsequent treatment. Interestingly, however, healthy and WS-affected coral samples in the present study were found to have very little to no impact on the growth of the reported coral pathogen (more specifically, previously proposed WS pathogen) *Vibrio harveyi*. This could suggest that antimicrobial capacity is unchanged in healthy and diseased specimens, with *E*. *lamellosa* appearing to have a naturally very limited antimicrobial capacity against *Vibrio harveyi*. This could suggest that not all corals, such as *Echinopora lamellosa*, possess such antimicrobial qualities, perhaps relying more on the physical defences of the mucus barrier. It is also possible that these corals are more susceptible to certain stresses and diseases, as observed across a range of corals in response to climate change with certain species found to be more susceptible than others [58, 59]. However, it could also be argued that the findings of this study support research that demonstrates different coral species have different antimicrobial selectivity. Selectivity is a hallmark of antimicrobial activity in many coral species [60–62] and it is possible that *E*. *lamellosa* features antimicrobial properties that do not select against *Vibrio harveyi*. To understand the overall antimicrobial capacity and selectivity of *E*. *lamellosa* (and other species), future antimicrobial assays using coral extracts should be carried out against a much broader range of target prokaryotes.

In the present study, all WS-affected samples (treated and untreated) were found to be host to a number of ciliate species, whilst no ciliates were identified in healthy *E*. *lamellosa* samples, thus making this the first study to identify and directly associate ciliates with WS in the plating coral *E*. *lamellosa*. A *Philaster* sp was also found to be present in all diseased samples in the present study, supporting previous studies associating this particular genus with the consumption of the corals *Symbiodinium* in WS, White Band Disease (WBD) and Brown Band Disease (BBD)[18, 29, 63]. The belief that this ciliate is responsible for the neat lesion edge associated with WS is supported by research into the role of another *Philaster* sp (*P*. *lucinda*) in WBD and WS [46, 63]. It was observed that when the ciliate was knocked down using metronidazole in WBD and WS samples, the morphology of lesion interface was altered, yet lesion progression continued. This promotes the theory that *P*. *lucinda* is a secondary agent in WBD, which could also be the case for *Philaster* species associated with WS. Other predominant ciliates found in the present study were *Cohnilembus* sp. and *Pseudokeronopsis* sp. There are no previous reports linking *Cohnilembus* sp. with corals and disease, despite this ciliate being found in all WS-affected *E*. *lamellosa* samples in the present study. *Cohnilembus* sp. is known to exist as a bacterivorous benthic suspension feeder which has enhanced feeding when attached to a surface, whereby stronger feeding currents can be created compared to when the ciliate is suspended in the water column [64]. It is possible therefore that during WS, *Cohnilembus* sp. attaches to areas of denuded skeleton that can no longer be protected by the host coral. A further ciliate species identified in this study, *Pseudokeronopsis* sp. was also found in WS and BBD samples by Sweet and Bythell [18], they observed this particular ciliate to be a ciliaphore, consuming other protists and predominantly found in areas of denuded skeleton. This indicates *Pseudokeronopsis* sp. does not have a role in WS pathology, but rather exists as part of a ciliate food chain colonising the exposed skeleton following the removal of tissue due to the disease. It remains uncertain whether the association of ciliates with WS is cause or effect, but it appears very clear that ciliates play an important role in the aetiology of WS lesions [29]. Interestingly, *Mesanophrys* spp., ciliates belonging to the order *Philasterida*, and morphologically similar to *Philaster*, have previously been associated with tissue degradation in different organisms, such as crabs [65, 66], lobsters [67] and fish (such as the turbot) [68]. Small, Neil [67] found that a *Mesanophrys* sp. isolated from infected lobsters express metalloproteases capable of proteolysis of several host proteins, including structural proteins such as myosin. We propose that future studies on coral disease should aim to determine the expression of similar proteases in coral disease-associated ciliates in order to determine if they are capable of direct host tissue stress and degradation, thus further clarifying their role in diseases such WS.

In conclusion, this study has determined shifts in the microbial rRNA gene profiles between healthy and WS-affected *E*. *lamellosa*. Given its association with the formation of lesions and ulceration, as well as tissue necrosis in various fish species, and based on its strong association with WS-affected samples in the present study, the bacterium *Tenacibaculum* sp. is presented as a novel potential causal agent for lesion formation and tissue necrosis in *Echinopora lamellosa*. Since this bacterium has not been reported as a causal agent for WS in the past, this supports the view that there is no specific causal agent for this disease, but rather various opportunistic necrotizing bacteria capable of tissue breakdown may have the capacity for initiating WS [29], thus the disease is likely polymicrobial. However, future studies focusing on fulfilling Koch’s postulates with regards to the role of *Tenacibaculum* sp in WS are required. This study further supports recent work showing that ciliates play an active role in disease morphology in many different species of coral, giving the disease its characterizing neat boundary at the lesion interface between healthy tissue and denuded skeleton. Future work should focus on the isolation of proteases and other molecules involved in tissue stress and degradation from associated ciliate isolates (particularly zooxanthellae consumers), thus determining whether or not they actively impair and break down healthy host tissue or whether they are passively feeding on already weakened tissue. Additionally, this study appears to demonstrate that not all corals possess probiotic qualities that aid in their defence against invasive microorgansims, which could contribute to why some corals are more susceptible to stress and disease than others.

## References

[1] Hayes RL, Goreau NI. The significance of emerging diseases in the tropical coral reef ecosystem. Revista de Biologia Tropical. 1998;46(5):173–85.

[2] Harvell CD. Emerging Marine Diseases—Climate Links and Anthropogenic Factors. Science. 1999;285(5433):1505–10. doi: 10.1126/science.285.5433.1505 10498537 10.1126/science.285.5433.1505

[3] Harvell CD, Mitchell CE, Ward JR, Altizer S, Dobson AP, Ostfeld RS, et al Climate warming and disease risks for terrestrial and marine biota. Science. 2002;296(5576):2158–62. doi: 10.1126/science.1063699 PubMed PMID: . 12077394 10.1126/science.1063699

[4] Rosenberg E, Ben-Haim Y. Microbial diseases of corals and global warming. Environmental Microbiology. 2002;4(6):319–26. 10.1046/j.1462-2920.2002.00302.x 12071977

[5] Pollock JS, Morris PJ, Willis BL, Bourne DG. The urgent need for robust coral disease diagnostics. PloS Pathogens. 2011;7(10). doi: 10.1371/ 10.1371/journal.ppat.1002183 PMC3197597 22028646

[6] Sokolow S. Effects of a changing climate on the dynamics of coral infectious disease: a review of the evidence. Diseases of Aquatic Organisms. 2009;87(1–2):5–18. doi: 10.3354/dao02109 20095237 10.3354/dao02099

[7] Piskorska M, Smith G, Weil E. Bacteria associated with the coral Echinopora lamellosa (Esper 1795) in the Indian Ocean—Zanzibar region. African Journal of Environmental Science and Technology. 2007;1(5):93–8.

[8] Sweet M, Jones R, Bythell J. Coral diseases in aquaria and in nature. Journal of the Marine Biological Association of the United Kingdom. 2011:1–11. doi: 10.1017/s0025315411001688

[9] Dalton SJ, Godwin S, Smith SDA, Pereg L. Australian subtropical white syndrome: a transmissible temperature-dependent coral disease. Marine and Freshwater Research. 2010;61:342–50.

[10] Sussman M, Willis BL, Victor S, Bourne DG. Coral pathogens identified for white syndrome (WS) epizootics in the Indo-Pacific. PLoS ONE. 2008;3(6):1–14. 10.1371/journal.pone.0002393 PMC2409975 18560584

[11] Bruno JF, Selig ER, Casey KS, Page CA, Willis BL, Drew Harvell C, et al Thermal stress and coral cover as drivers of coral disease outbreaks. PLoS Biol. 2007;5(6):1220–7. 10.1371/journal.pbio.0050124 PMC1865563 17488183

[12] Aeby GS, Ross M, Williams GJ, Lewis TD, Work TM. Disease dynamics of Montipora white syndrome within Kaneohe Bay, Oahu, Hawaii: distribution, seasonality, virulence, and transmissibility. Diseases of Aquatic Oragnisms. 2010;91:1–8. 10.3354/dao02247 20853736

[13] Willis BL, Page CA, Dinsdale EA. Coral disease on the Great Barrier Reef In: Rosenberg E, Loya Y, editors. Coral Health and Disease: Springer, Berlin; 2004 p. 69–104.

[14] Luna GM, Bongiorni L, Gili C, Biavasco F, Danovaro R. Vibrio harveyi as a causative agent of the White Syndrome in tropical stony corals. Environmental Microbiology Reports. 2010;2(1):120–7. doi: 10.1111/j.1758-2229.2009.00114.x 23766006 10.1111/j.1758-2229.2009.00114.x

[15] Ushijima B, Smith A, Aeby GS, Callahan SM. *Vibrio owensii* Induces the Tissue Loss Disease *Montipora* White Syndrome in the Hawaiian Reef Coral *Montipora capitata* . PLoS ONE. 2012;7(10):e46717 doi: 10.1371/journal.pone.0046717.g001 23056419 10.1371/journal.pone.0046717 PMC3466290

[16] Wagner M, Amann R, Lemmer H, Schleifer K. Probing activated sludge with oligonucleotides specific for proteobacteria: inadequacy of culture-dependent methods for describing microbial community structure. Applied and Environmental Microbiology. 1993;59(5):1520–5. 8517747 10.1128/aem.59.5.1520-1525.1993 PMC182113

[17] Scanlan PD, Marchesi JR. Micro-eukaryotic diversity of the human distal gut microbiota: qualitative assessment using culture-dependent and -independent analysis of faeces. ISME J. 2008;2(12):1183–93. doi: 10.1038/ismej.2008.76 PubMed PMID: . 18670396 10.1038/ismej.2008.76

[18] Sweet M, Bythell J. Ciliate and bacterial communities associated with White Syndrome and Brown Band Disease in reef-building corals. Environmental Microbiology. 2012. doi: 10.1111/j.1462-2920.2012.02746.x 10.1111/j.1462-2920.2012.02746.x PMC3465780 22507379

[19] Sussman M, Mieog JC, Doyle J, Victor S, Willis BL, Bourne DG. Vibrio zinc-metalloprotease causes photoinactivation of coral endosymbionts and coral tissue lesions. PLoS ONE. 2009;4(2):1–14. doi: 10.1371/journal.pone.0004511.t001 10.1371/journal.pone.0004511.t002 10.1371/journal.pone.0004511 PMC2637982 19225559

[20] Rosenberg E, Koren O, Reshef L, Efrony R, Zilber-Rosenberg I. The role of microorganisms in coral health, disease and evolution. Nature Reviews Microbiology. 2007;5(5):355–62. doi: 10.1038/nrmicro1635 17384666 10.1038/nrmicro1635

[21] Reshef L, Koren O, Loya Y, Zilber-Rosenberg I, Rosenberg E. The Coral Probiotic Hypothesis. Environmental Microbiology. 2006;8(12):2068–73. doi: 10.1111/j.1462-2920.2006.01148.x 17107548 10.1111/j.1462-2920.2006.01148.x

[22] Nissimov J, Rosenberg E, Munn CB. Antimicrobial properties of resident coral mucus bacteria of Oculina patagonica. FEMS microbiology letters. 2009;292(2):210–5. Epub 2009/02/05. doi: 10.1111/j.1574-6968.2009.01490.x PubMed PMID: . 19191871 10.1111/j.1574-6968.2009.01490.x

[23] Shnit-Orland M, Kushmaro A. Coral mucus-associated bacteria: a possible first line of defense. FEMS Microbiology Ecology. 2009;67(3):371–80. doi: 10.1111/j.1574-6941.2008.00644.x 19161430 10.1111/j.1574-6941.2008.00644.x

[24] Kvennefors ECE, Sampayo E, Kerr C, Vieira G, Roff G, Barnes AC. Regulation of Bacterial Communities Through Antimicrobial Activity by the Coral Holobiont. Microbial ecology. 2012;63(3):605–18. doi: 10.1007/s00248-011-9946-0 21984347 10.1007/s00248-011-9946-0

[25] Kumar KS, Haritha R, Mohan YSYJ, Ramana T. Screening of marine actinobacteria for antimicrobial compounds. Research Journal of Microbiology. 2011;6(4):385–93.

[26] Kennedy J, Baker P, Piper C, Cotter PD, Walsh M, Mooij MJ, et al Isolation and analysis of bacteria with antimicrobial activities from the marine sponge Haliclona simulans collected from Irish waters. Mar Biotechnol (NY). 2009;11(3):384–96. doi: 10.1007/s10126-008-9154-1 PubMed PMID: . 18953608 10.1007/s10126-008-9154-1

[27] Manivasagan P, Gnanam S, Sivakumar K, Thangaradjou T, Vijayalakshmi S, Balasubramanian T. Antimicrobial and cytotoxic activities of an actinobacteria *(Streptomyces sp*. PM-32) isolated from an offshore sediments of the Bay of Bengal in Tamilnadu. Advances in Biological Research. 2009;3(5–6):231–6.

[28] Sweet MJ, Smith D, Bythell JC, Craggs J. Changes in microbial diversity associated with two coral species recovering from a stressed state in a public aquarium. Journal of Zoo and Aquarium Research. 2013;1(2):1–8.

[29] Sweet MJ, Craggs J, Robson J, Bythell JC. Assessment of the microbial communities associated with white syndrome and brown jelly syndrome in aquarium corals. Journal of Zoo and Aquarium Research. 2013;1(1):1–8.

[30] Harvell D, Jordán-Dahlgren E, Merkel S, Rosenberg E, Raymundo L, Smith G, et al Coral disease, environmental drivers, and the balance between coral and microbial associates. Oceanography. 2007;20(1):172–95.

[31] Muyzer G, Smalla K. Application of denaturing gradient gel electrophoresis (DGGE) and temperature gradient gel electrophoresis (TGGE) in microbial ecology. Antonie van Leeuwenhoek. 1998;73:127–41. 9602286 10.1023/a:1000669317571

[32] Jousset A, Lara E, Nikolausz M, Harms H, Chatzinotas A. Application of the denaturing gradient gel electrophoresis (DGGE) technique as an efficient diagnostic tool for ciliate communities in soil. The Science of the total environment. 2010;408(5):1221–5. doi: 10.1016/j.scitotenv.2009.09.056 PubMed PMID: . 19896703 10.1016/j.scitotenv.2009.09.056

[33] Janse I, Bok J, Zwart G. A simple remedy against artifactual double bands in denaturing gradient gel electrophoresis. Journal of microbiological methods. 2004;57(2):279–81. doi: 10.1016/j.mimet.2003.12.006 PubMed PMID: . 15063068 10.1016/j.mimet.2003.12.006

[34] Bourne DG, Boyett HV, Henderson ME, Muirhead A, Willis BL. Identification of a ciliate (Oligohymenophorea: Scuticociliatia) associated with brown band disease on corals of the Great Barrier Reef. Appl Environ Microbiol. 2008;74(3):883–8. doi: 10.1128/AEM.01124-07 PubMed PMID: ; PubMed Central PMCID: PMC2227702. 18083868 10.1128/AEM.01124-07 PMC2227702

[35] Sweet MJ, Croquer A, Bythell JC. Temporal and spatial patterns in waterborne bacterial communities of an island reef system. Aquatic Microbial Ecology. 2010;61(1):1–11.

[36] Selinummi J, Seppälä J, Yli-Harja O, Puhakka J. Software for quantification of labeled bacteria from digital microscope images by automated image analysis. BioTechniques. 2005;39(6):859–63. doi: 10.2144/000112018 16382904 10.2144/000112018

[37] Sweet MJ, Croquer A, Bythell JC. Dynamics of bacterial community development in the reef coral Acropora muricata following experimental antibiotic treatment. Coral Reefs. 2011;30(4):1121–33. doi: 10.1007/s00338–011–0800–0

[38] Clarke KR, Warwick RM. A further biodiversity index applicable to species lists—variation in taxonomic distinctness. Marine Ecology Progress Series. 2001;216:265–78.

[39] Cróquer A, Bastidas C, Elliott A, Sweet M. Bacterial assemblages shifts from healthy to yellow band disease states in the dominant reef coral *Montastraea faveolata* . Environmental Microbiology Reports. 2012 doi: 10.1111/j.1758-2229.2012.00397.x 10.1111/j.1758-2229.2012.00397.x 23757136

[40] Dereeper A, Guignon V, Blanc G, Audic S, Buffet S, Chevenet F, et al Phylogeny. fr: robust phylogenetic analysis for the non-specialist. Nucleic acids research. 2008;36(suppl 2):W465–W9. 18424797 10.1093/nar/gkn180 PMC2447785

[41] Sunagawa S, DeSantis TZ, Piceno YM, Brodie EL, DeSalvo MK, Voolstra CR, et al Bacterial diversity and White Plague Disease-associated community changes in the Caribbean coral Montastraea faveolata. The ISME Journal. 2009;3(5):512–21. doi: 10.1038/ismej.2008.131 19129866 10.1038/ismej.2008.131

[42] Avendano-Herrera R, Toranzo AE, Magarinos B. Tenacibaculosis infection in marine fish caused by Tenacibaculum maritimum: a review. Diseases of Aquatic Oragnisms. 2006;71:255–66. 17058606 10.3354/dao071255

[43] Harder T, Lau SCK, Dobretsov S, Fang TK, Qian P. A distinctive epibiotic bacterial community of the soft coral *Dendronephthya sp* and antibacterial activity of coral tissue extracts suggest a chemical mechanism against bacterial epibiosis. FEMS Microbiology Ecology. 2008;43:337–47. 10.1111/j.1574-6941.2003.tb01074.x 19719665

[44] El-Galil MAA, Hashem M. Epidemiological and bacteriological studies on tenacibaculosis in some Red Sea fishes, Egypt. International Journal of Environmental Science and Engineering. 2012;3:25–32.

[45] Smith D, Leary P, Bendall M, Flach E, Jones R, Sweet M. A Novel Investigation of a Blister-Like Syndrome in Aquarium Echinopora lamellosa. PloS one. 2014;9(5):e97018 doi: 10.1371/journal.pone.0097018 24827734 10.1371/journal.pone.0097018 PMC4020768

[46] Sweet M, Bythell J. White Syndrome in Acropora muricata: Non‐specific bacterial infection and ciliate histophagy. Molecular ecology. 2015;In Press. 10.1111/mec.13097 PMC4964940 25652762

[47] D'Alvise PW, Lillebø S, Wergeland HI, Gram L, Bergh Ø. Protection of cod larvae from vibriosis by Phaeobacter spp.: A comparison of strains and introduction times. Aquaculture. 2013;384–387:82–6. doi: 10.1016/j.aquaculture.2012.12.013

[48] Prado S, Montes J, Romalde JL, Barja JL. Inhibitory activity of *Phaeobacter* strains against aquaculture pathogenic bacteria. International Microbiology. 2009;12:107–14. doi: 10.2436/20.1501.01.87 19784930

[49] Porsby CH, Nielsen KF, Gram L. *Phaeobacter* and *Ruegeria* species of the *Roseobacter* clade colonize separate niches in a Danish Turbot (*Scophthalmus maximus*)-rearing farm and antagonize *Vibrio anguillarum* under different growth conditions. Applied Environmental Microbiology. 2008;74(23):7356–64. doi: 10.1128/AEM.01738-08 PubMed PMID: ; PubMed Central PMCID: PMC2592939. 18952864 10.1128/AEM.01738-08 PMC2592939

[50] D'Alvise PW, Lillebo S, Prol-Garcia MJ, Wergeland HI, Nielsen KF, Bergh O, et al *Phaeobacter gallaeciensis* reduces *Vibrio anguillarum* in cultures of microalgae and rotifers, and prevents vibriosis in cod larvae. PLoS One. 2012;7(8):e43996 doi: 10.1371/journal.pone.0043996 PubMed PMID: ; PubMed Central PMCID: PMC3425499. 22928051 10.1371/journal.pone.0043996 PMC3425499

[51] Radjasa OK, Sabdono A. Ecological role of a softcoral-associated bacterium *Arthrobacter* sp. on marine biofilm-forming bacteria. Microbiology Indonesia. 2008;2(2):84–8.

[52] Shivvers DW, Brock TD. Oxidation of elemental sulfur by *Sulfolobus acidocaldarius* . Journal of Bacteriology. 1973;114(2):706–10. 4706192 10.1128/jb.114.2.706-710.1973 PMC251830

[53] Brock TD, Brock KM, Belly RT, Weiss RL. *Sulfolobus*: A new genus of sulfur-oxidizing bacteria living at low pH and high temperature. Archives of Microbiology. 1972;84:54–68. 4559703 10.1007/BF00408082

[54] Sato Y, Willis BL, Bourne DG. Successional changes in bacterial communities during the development of black band disease on the reef coral, *Montipora hispida* . ISME J. 2010;4(2):203–14. Epub 2009/09/25. doi: 10.1038/ismej.2009.103 PubMed PMID: . 19776765 10.1038/ismej.2009.103

[55] Raina JB, Tapiolas D, Willis BL, Bourne DG. Coral-associated bacteria and their role in the biogeochemical cycling of sulfur. Appl Environ Microbiol. 2009;75(11):3492–501. Epub 2009/04/07. doi: 10.1128/AEM.02567-08 PubMed PMID: ; PubMed Central PMCID: PMC2687302. 19346350 10.1128/AEM.02567-08 PMC2687302

[56] Muscantine L, Falkowski PG, Dubinsky Z, Cook PA, McCloskey LR. The effect of external nutrient resources on the population dynamics of zooxanthellae in a reef coral. Proceedings of the Royal Society B: Biological Sciences. 1989;236:311–24. 2760652

[57] Luna GM, Biavasco F, Danovaro R. Bacteria associated with the rapid tissue necrosis of stony corals. Environ Microbiol. 2007;9(7):1851–7. doi: 10.1111/j.1462-2920.2007.01287.x PubMed PMID: . 17564618 10.1111/j.1462-2920.2007.01287.x

[58] Foden W, Mace GM, Vié J-C, Angulo A, Butchart SHM, DeVantier L, et al. Species susceptibility to climate change impacts. Wildlife in a changing world–an analysis of the 2008 IUCN Red List of threatened species2009. p. 77.

[59] Harvell CD, Altizer S, Cattadori IM, Harrington L, Weil E. Climate change and wildlife diseases: when does the host matter the most? Ecology. 2009;90(4):912–20. 19449685 10.1890/08-0616.1

[60] Gunthorpe L, Cameron AM. Widespread but variable toxicity in scleractinian corals. Toxicon. 1990;28(10):1199–219. 1979891 10.1016/0041-0101(90)90120-v

[61] Gochfeld DJ, Pappas KE, Lee S, Aeby GS. Variability in Antibacterial Activity in Hawaiian Corals. 2011.

[62] Marquis CP, Baird AH, De Nys R, Holmström C, Koziumi N. An evaluation of the antimicrobial properties of the eggs of 11 species of scleractinian corals. Coral Reefs. 2005;24(2):248–53.

[63] Sweet MJ, Croquer A, Bythell JC. Experimental antibiotic treatment identifies potential pathogens of white band disease in the endangered Caribbean coral Acropora cervicornis. Proceedings of the Royal Society of London B: Biological Sciences. 2014;281(1788):20140094 doi: 10.1098/rspb.2014.0094 24943374 10.1098/rspb.2014.0094 PMC4083779

[64] Shimeta J, Starczak VR, Ashiru OM, Zimmer CA. Influences of benthic boundary-layer flow on feeding rates of ciliates and flagellates at the sediment-water interface. Limnology and oceanography. 2001;46(7):1709–19.

[65] Morado JF, Giesecke RH, Syrjala SE. Molt related mortalities of the Dungeness crab Cancer magister caused by a marine facultative ciliate Mesanophrys pugettensis. Diseases of aquatic organisms. 1999;38(2):143–50.

[66] Messick GA, Small EB. Mesanophrys chesapeakensis n. sp., a histophagous ciliate in the blue crab, Callinectes sapidus, and associated histopathology. Invertebrate Biology. 1996:1–12.

[67] Small HJ, Neil DM, Taylor AC, Coombs GH. Identification and partial characterisation of metalloproteases secreted by a Mesanophrys-like ciliate parasite of the Norway lobster Nephrops norvegicus. Diseases of Aquatic Organisms. 2005;67:225–31. 16408838 10.3354/dao067225

[68] Qin L, Wang Y, Zhang L, Dai J. Histopathology of turbot associated with Mesanophrys carcini parasite. Acta Hydrobiologica Sinica. 2007;31(5):622.

